# Neurological Peculiarities of POEMS Syndrome: Experience From a Brazilian University Center

**DOI:** 10.1002/mus.70114

**Published:** 2025-12-18

**Authors:** Renan Fabri Rosenstein, Jose Pedro Soares Baima, Thales Dalessandro Meneguin Pereira, Gracia Aparecida Martinez, Carlos Otto Heise, Angelina Maria Martins Lino

**Affiliations:** ^1^ Divisão de Clínica Neurológica Hospital das Clínicas da Faculdade de Medicina da Universidade de São Paulo‐São Paulo São Paulo Brazil; ^2^ Unidade Do Sistema Nervoso Hospital Universitário da Universidade Federal Do Ceará Fortaleza Ceará Brazil; ^3^ Instituto de Câncer do Hospital das Clínicas da Faculdade de Medicina da Universidade de São Paulo‐São Paulo São Paulo Brazil; ^4^ Divisão de Hematologia Hospital das Clínicas da Faculdade de Medicina da Universidade de São Paulo‐São Paulo São Paulo Brazil

**Keywords:** chronic inflammatory demyelinating polyradiculoneuropathy, nerve conduction studies, plasma cell neoplasm, POEMS syndrome, polyneuropathies

## Abstract

**Aim:**

Polyneuropathy, organomegaly, endocrinopathy, monoclonal protein, and skin changes (POEMS) syndrome is a rare paraneoplastic syndrome associated with significant neurologic morbidity. Better understanding of the manifestations of this disease is crucial to early diagnosis and improvement of prognosis.

**Methods:**

We retrospectively reviewed medical record data of adult patients diagnosed with POEMS syndrome between 2007 and December 2023 fulfilling the 2014 International Myeloma Working Group criteria for POEMS syndrome in a single tertiary care Hospital in Brazil. Clinical, laboratory and electrophysiological data were analyzed.

**Results:**

Thirty‐three patients were included, with median time from symptom onset to diagnosis of 13 (11–35) months, and 30% of patients were nonambulatory at the diagnosis. Neuropathy was present in 100% of patients, and early proximal muscle weakness was present in 57%. Nerve conductions studies (NCS) disclosed demyelinating polyneuropathy with axonal damage in 96% and conduction block in 33%. Serum monoclonal paraprotein heavy chain was IgG subtype in 73% and lambda light chain in 91%. Castleman disease was diagnosed in 6% of patients. Organomegaly, bone lesions and endocrinological profiles were similar to previously reported cohorts.

**Discussion:**

Patients in our cohort had similar clinical profiles to previously reported data. However, unlike other large cohorts, our study showed a high proportion of IgG class paraprotein, non‐length‐dependent findings and conduction block on electrophysiology, and a lower number of cases associated with Castleman's disease. Our findings highlight the importance of pursuing a POEMS syndrome diagnosis even in the presence of atypical electrophysiological features.

**Abbreviations**
CB: conduction block
CIDP: chronic immune demyelinating polyradiculoneuropathy
CMAP: compound motor action potential
CSF: cerebrospinal fluid
EAN: European Academy of Neurology
IgA: immunoglobulin A
IgG: immunoglobulin G
IgM: immunoglobulin M
MRI: magnetic resonance imaging
NCS: nerve conduction studies
PET: positron emission tomography
POEMS: polyneuropathy, organomegaly, endocrinopathy, monoclonal gammopathy, and skin changes
SNAP: sensory nerve action potential
SPE: serum plasma electrophoresis
TD: temporal dispersion
VEGF: vascular endothelial growth factor

## 1 Introduction

POEMS (Polyneuropathy, Organomegaly, Endocrinopathy, Monoclonal protein, Skin changes) syndrome is a condition in which the presence of a monoclonal population of plasma cells leads to myriad manifestations in various organs and systems.

The syndrome is considered rare. An epidemiological study in Japan estimated its prevalence at 0.3 per 100,000 people [1]. If not recognized, most patients will develop quadriparesis and systemic manifestations with an impact on quality of life and productivity [2]. Survival in patients with POEMS syndrome has improved from a median of 33 months in the 1980s [3] to 165 months in the 2000s with the development of new chemotherapeutics and the advent of autologous stem cell transplantation. Most deaths occur due to disease progression or systemic, particularly cardiopulmonary, complications.

Neuropathy is a mandatory criterion for the diagnosis of the disease, and early diagnosis is of paramount importance to the prognosis of patients with POEMS. Clinical, laboratory and electrophysiological characteristics of peripheral nervous system involvement are useful in differentiating POEMS syndrome from other neuropathies, mainly chronic inflammatory demyelinating polyradiculoneuropathy (CIDP).

Large cohorts describing the disease have been previously published [1, 2, 4, 5, 6, 7], but South American reports are limited to individual case reports and small series [8, 9]. Clinical and laboratory idiosyncrasies of the disease appear to exist across different regions, and the same may apply to Brazilian patients.

The aim of this study is to describe the clinical profile of Brazilian patients with this syndrome.

## 2 Methods

We retrospectively reviewed the medical records of 33 patients diagnosed with POEMS syndrome, identified among 941 individuals evaluated at the Peripheral Nerve Outpatient Clinic of the Hospital das Clínicas da Faculdade de Medicina da USP—São Paulo, Brazil between January 2007 and October 2023. Eligible patients were ≥ 18 years old and met the 2014 International Myeloma Working Group diagnostic criteria for POEMS syndrome [10]. Other potential causes of neuropathy, such as severe alcoholism [11], prior chemotherapy, vitamin deficiency, or other metabolic abnormalities were excluded.

Data collection included demographics (sex, age), the time from first symptom onset to diagnosis, and a detailed list of clinical manifestations. Neuropathic and autonomic symptoms, organomegaly, endocrinopathy, and skin changes were all documented. Laboratory findings included hematological and endocrinological markers, cerebrospinal fluid (CSF) analysis, and vascular endothelial growth factor (VEGF) levels when available. The types of bone lesions were also recorded from imaging studies. Neuropathic symptoms were quantified using the modified polyneuropathy disability score [12].

Electrophysiological studies were performed under the supervision of board‐certified electrophysiologists using standard protocols. To ensure procedural consistency, we included only examinations performed at our institution at the time of diagnosis. Motor nerve conduction studies (NCS) were recorded from the median, ulnar, peroneal, and tibial nerves. Sensory NCS were recorded from the median, ulnar, superficial radial, sural, and superficial peroneal nerves. All electrodiagnostic studies were reviewed for fulfillment of demyelination and conduction block (CB) criteria established by the European Academy of Neurology/Peripheral Nerve Society (EAN/PNS) in 2021 [13]. Axonal loss was defined as a reduction in the amplitude of the compound motor action potential (CMAP) or sensory nerve action potential (SNAP) compared with our institutional reference values. For all motor nerves with a recordable CMAP, the terminal latency index (TLI) was determined according to the formula TLI = *D*/(MNCV × DML), with *D* indicating the distal conduction distance (mm), MNCV the motor nerve conduction velocity (m/s), and DML the distal motor latency (ms) [14].

## 3 Results

Thirty‐three patients with a diagnosis of POEMS syndrome were included, of whom 22 (67%) were men. The median age at diagnosis was 51 (44–57) years and the median time from first symptom to diagnosis was 13 (11–35) months. The median follow‐up was 60 (1–184) months, during which 11 patients (33%) died. The most frequent causes of death were disease progression and cardiopulmonary failure.

### 3.1 Polyneuropathy Manifestations

As expected, polyneuropathy was present in all patients. It was sensorimotor in 82%, while the remaining 18% exhibited purely sensory symptoms on clinical assessment, with motor involvement detected only by NCS (Table 1). The most common sensory symptoms were paresthesia (90%) and pain (54%). Autonomic dysfunction was rare, occurring in only one patient, who reported erectile dysfunction and orthostatic hypotension.

**TABLE 1:** Motor status at diagnosis using the modified peripheral nerve disability score.

Asymptomatic	0%
Sensory symptoms, walking normally	18%
Walking difficulties, not requiring support	24%
Needs one stick for ambulation	9%
Needs walking frame	18%
Confined to bed or wheelchair	30%

*Source*: Adapted from Ando et al. [12].

Although neuropathy followed a length‐dependent pattern in 85% of cases, 57% of patients exhibited early proximal muscle weakness and gait impairment, resembling a polyradiculoneuropathy. Among these, one patient displayed an acute‐CIDP–like phenotype with symmetric quadriparesis and bilateral facial palsy, initially improving but subsequently worsening beyond 8 weeks from symptom onset. Cerebrospinal fluid (CSF) analysis, performed in 43% of patients, revealed protein–cytologic dissociation in 93% of samples.

### 3.2 Electrophysiological Findings

At diagnosis, 24 patients underwent NCS at our institution (Table 2). NCS from the nine remaining patients were excluded from the analysis because they were performed at other centers, despite confirmation of sensorimotor polyneuropathy. Median time from initial neuropathy symptoms and NCS was 10.8 (7.8–21.6) months.

**TABLE 2:** Neurophysiological characteristics in 24 patients with POEMS syndrome.

SNAP amplitude^a^ (μV)	Median	Range	Reference value (μV)	% Abnormal
Median	8	0–47.7	> 20	75
Ulnar	5.9	0–52.3	> 10	58
Radial	11	0–33.9	> 10	47
Peroneal	0	0–13	> 5	88
Sural	0	0–9	> 6	94

CMAP amplitude^a^ (mV)	Median	Range	Reference value (mV)	% Abnormal
Median	7.7	0–17	> 5	28
Ulnar	5.8	0.7–21	> 6	53
Peroneal	0	0–3	> 2	93
Tibial	0	0–3.1	> 6	100

Motor conduction velocity (m/s)	Median	Range	Reference value (m/s)	% Abnormal
Median	31.6	10.5–55.5	> 50	94
Ulnar	29.8	10–52.2	> 50	89
Peroneal	37	25–41.1	> 42	100
Tibial	31.2	18.7–41.9	> 42	100

*F* wave latency (ms)	Median	Range	Reference value (ms)	% Abnormal
Median	44.2	29.8–60.4	> 2 SD according to height	88
Ulnar	41.4	28.4–69.2	> 2 SD according to height	80
Peroneal	60.5	60.2–60.8	> 2 SD according to height	100
Tibial	69.3	64.1–77.5	> 2 SD according to height	100

Abbreviations: μV, micro Volt; CMAP, compound motor action potential; m/s, meter per second; ms, milliseconds; mV, millivolt; SD, standard deviation; SNAP, sensory nerve action potential.

^a^ Amplitude measured from peak‐to‐peak.

The electrophysiological EAN/PNS criteria for CIDP [13] were met in 92%. The mean motor conduction velocity (CV) was reduced, being 33.9 and 33.8 m/s in upper and lower limbs, respectively. A high rate of axonal damage was evident, with CMAPs being unrecordable in lower limb nerves in 66% of patients. SNAPs were absent in all nerves in 21% of patients and in the lower limbs in 71%. A notable finding was the high rate of conduction block (CB), observed in 33% of our patients. CB was detected in seven ulnar nerves in the forearm (47%), in seven median nerves in the forearm (47%), and in a fibular nerve in the leg (7%). Martin‐Gruber anastomosis was excluded in all patients with ulnar CB. Temporal dispersion (TD) occurred in five patients (21%) and the co‐occurrence of CB and TD findings were observed in two patients (8%). The TLI was ≥ 0.25 in all nerves in which a CMAP was obtained. Median nerve TLI was > 0.38 in 59% of patients. Sural sparing was not found in any NCS. At least 54% of patients satisfied both clinical and electrophysiological criteria for CIDP at time of diagnosis.

### 3.3 Plasma Cell Disorder

All patients met the mandatory criterion of a monoclonal plasma cell disorder. A serum monoclonal spike was detected by serum protein electrophoresis (SPE) in 39% of patients and by serum immunofixation (SIF) in 79%. The predominant paraprotein was IgG‐λ (Table 3). In seven patients (21%) with negative SPE and SIF, the plasma cell disorder was diagnosed by bone biopsy. Bone marrow biopsy and/or aspirate was performed in 32 patients (97%) at diagnosis, revealing > 10% plasma cell infiltration in 52%.

**TABLE 3:** Relative frequency of clinical, imaging and laboratory findings of major criteria in POEMS syndrome in different studies.

Clinical features	Current study *n* = 33	Keddie et al. [4] *n* = 100	Suichi et al. [1] *n* = 167	Li et al. [6] *n* = 99	Lee‐Chen et al. [28] *n* = 11
Peripheral neuropathy	100	96	100	98	100
Raised CSF protein^a^	93 (13/14)	100	NA	96	NA
Monoclonal plasma cell disorder	100	100	89	99	100
Serum heavy chain subtype					
IgM^b^	0	0	1	0	9
IgG^b^	73	45	52	24	72
IgA^b^	27	53	47	72	18
Lambda	91	98	92	93	54
Kappa	9	2	8	3	46
Castleman disease	6	14	11	58	27
Bone lesions	78	81	58	27	63
Solitary lesion^c^	50	38	34	NA	NA
Sclerotic^c^	58	55	81	70	71
Lytic^c^	11	15	7	30	0
Mixed^c^	31	30	11	0	29
Vascular endothelial growth factor elevation^d^	NR (4/5)	94	86	70	NA

Abbreviations: CSF, cerebrospinal fluid; Ig, immunoglobulin; *n*, number; NA, information not available; NR, not representative; VEGF, vascular endothelial growth factor.

^a^ CSF protein was considered elevated if ≥ 50 mg/dL for adults ≤ 50 years and ≥ 60 mg/dL if > 50 years.

^b^ Percentage of patients with abnormal serum immunofixation.

^c^ Percentage of patients with bone lesions.

^d^ VEGF was considered elevated if value > 1000 pg/mL in serum or > 146 pg/mL in plasma.

### 3.4 Major Criteria

Bone lesions, mostly sclerotic type, were present in 78% of patients and detected by computed tomography in 68%, positron emission tomography in 3%, magnetic resonance imaging in 21%, and scintigraphy in 7%. Only two patients (6%) were diagnosed with Castleman disease. Of the five patients who underwent VEGF measurement, titers were elevated in four.

### 3.5 Minor Criteria

Endocrine dysfunction was found in 23 (70%) patients, most commonly hypogonadism and hypothyroidism. Skin changes and extravascular volume overload were also prevalent. The frequencies of other minor criteria are presented in Table S1. Lymphadenopathy occurred in nine patients. Of the six biopsied, two had Castleman disease and four showed follicular hyperplasia without evidence of infection or autoimmunity. The remaining three were not biopsied due to inaccessibility, but met other POEMS syndrome criteria.

## 4 Discussion

Brazilian patients with POEMS syndrome show broadly similar features to other cohorts, with a few notable exceptions. Our cohort's demographic profile was consistent with that of previously described POEMS cohorts [1, 2, 4, 5, 15]. The mean diagnostic delay exceeded 1 year, similar to earlier reports and likely reflecting structural barriers within the Brazilian public healthcare system, including limited access to tertiary care and the unavailability of VEGF testing. This delay, along with poor performance status at diagnosis, restricted availability of modern chemotherapeutic agents for plasma cell dyscrasias, and postponed treatment initiation, may have contributed to the high mortality rate observed in our cohort, which is similar to older reports [16] but substantially higher than contemporary series, which report 5‐year survival rates exceeding 90% [17].

Our findings highlight a critical diagnostic pitfall in the clinical presentation of neuropathy in POEMS syndrome. While classically regarded as length‐dependent [4], neuropathy in our cohort frequently manifested with early proximal weakness and gait impairment, consistent with a non‐length‐dependent distribution that may resemble chronic, or even acute, demyelinating polyradiculoneuropathy. This pattern has also been described in other case reports [18, 19]. This observation is clinically relevant, as it underscores the need to consider POEMS syndrome in the differential diagnosis of atypical demyelinating neuropathies, potentially enabling earlier diagnosis. In line with previous reports, autonomic involvement was uncommon [20], whereas pain was frequent, supporting its role as a clinical clue for distinguishing POEMS from CIDP. These findings highlight the need to investigate all cases of peripheral neuropathy for paraproteinemia [21].

The high rates of conduction block (CB) and temporal dispersion (TD) observed in our electrophysiological analysis were also noteworthy. Reported in approximately 6% of POEMS patients [4, 22, 23], CB is generally considered rare and atypical, although a recent study documented it in 25% of tested nerves and 86% of patients [24]. Differences in diagnostic yield are unlikely to be explained solely by variations in CB criteria and may also reflect factors such as the timing of NCS relative to symptom onset, the number of nerves tested, and the rigor with which CB was investigated. Considering the disease stage at which NCS were performed, it is plausible to hypothesize the existence of reversible CB, in line with recent reports of Neurofascin‐155 and ‐140 antibodies in POEMS [25]. Importantly, the presence of CB or TD should not be used to exclude the diagnosis, particularly in patients with supportive clinical features.

TLI values < 0.25 have been reported as markers of pronounced distal conduction slowing and can differentiate anti‐MAG neuropathy from typical CIDP with high accuracy [26, 27]. Conversely, values > 0.38 in the median nerves demonstrated 70% sensitivity and 77% specificity in distinguishing POEMS from typical CIDP [22], proving particularly useful before the onset of the characteristic multi‐organ involvement of POEMS [28].

In line with previous studies, most patients exhibited a monoclonal lambda light chain. Unlike the slight IgA predominance typically reported, IgG was nearly three times more frequent than IgA in our cohort [1, 2, 4, 6], a pattern also observed in another small Latin American series [29]. Further studies are needed to determine whether this finding reflects a peculiarity of our population or merely results from the smaller sample size.

Only two patients in our sample were diagnosed with Castleman disease, despite extensive investigation in patients with confirmed lymphadenopathy. This rate is lower than that observed in other series. This finding is relevant because neuropathy in patients with POEMS syndrome associated with Castleman disease is typically milder and sensory‐predominant [30], and may partially explain the high rate of functional impairment in our sample.

The rates of organomegaly, extravascular volume overload, and cutaneous lesions in our cohort were comparable to those reported in other series. Systemic findings remain an important diagnostic clue for POEMS syndrome in the diagnostic evaluation of neuropathy. Central nervous system involvement is also common, typically presenting as stroke or pachymeningitis, which is often asymptomatic and has been reported in up to 71% of patients [4, 31].

The main limitations of this study are the retrospective design, and a small sample size compared to other published series. Furthermore, this study did not target data on treatment and prognosis. Finally, it is possible that some of our findings are due to underdiagnosis of the disease in our setting, partly due to the unavailability of VEGF in the Brazilian public health system.

## 5 Conclusions

In comparison to other reported series, our study of Brazilian patients with POEMS syndrome revealed a higher rate of CIDP‐like presentations, a lower proportion of Castleman's disease, and a different heavy‐chain paraprotein profile. Nerve conduction studies often met the criteria for CIDP, with early and significant axonal loss. However, the higher‐than‐expected rates of conduction block and temporal dispersion highlight that these features should not preclude a POEMS diagnosis.

This study underscores the critical importance of screening all patients with suspected CIDP for paraproteinemic diseases, particularly in a setting in which diagnostic tools may be limited. Early recognition of these unique clinical and electrophysiological patterns is key to a timely diagnosis, which is the cornerstone for improving patient outcomes.

## Author Contributions

**Renan Fabri Rosenstein:** investigation, conceptualization, writing – original draft, writing – review and editing, formal analysis.

## Funding

The authors have nothing to report.

## Ethics Statement

We confirm that we have read the Journal's position on issues involved in ethical publication and affirm that this report is consistent with those guidelines. This study was approved by the ethics committee of our institution, inscribed under protocol number 64106022.4.0000.0068.

## Conflicts of Interest

The authors declare no conflicts of interest.

## Supporting information


**Table S1:** Relative frequency of clinical, imaging and laboratory findings of minor criteria in POEMS syndrome in different studies.

## Data Availability

The data that support the findings of this study are available on request from the corresponding author. The data are not publicly available due to privacy or ethical restrictions.
