# Supplementary material for: Neurological Peculiarities of POEMS Syndrome: Experience From a Brazilian University Center
Source: Muscle Nerve. 2025 Dec 18;73(3):421–6. doi: 10.1002/mus.70114 (PMC12888833; doi:10.1002/mus.70114)
Supplement: Supplementary file 1 — Table S1: Relative frequency of clinical, imaging and laboratory findings of minor criteria in POEMS syndrome in different studies. [file MUS-73-421-s001.docx]

**Supplementary Table 1:** Relative frequency of clinical, imaging and laboratory findings of minor criteria in POEMS syndrome in different studies

| **Clinical features** | **Current study**  n=33 | **Keddie et al. 2020 [4]**  n=100 | **Suichi et al. 2019 [1]**  n=167 | **Li et al. 2011 [6]**  n=99 | **Lee-Chen, 2021 [28]**  n=11 |
| --- | --- | --- | --- | --- | --- |
| **Endocrine abnormality** | 70 | 68 | 65 | NA | 100 |
| **Gonadal axis** | 48 | 49 | 21 | 40 | 54 |
| **Adrenal axis** | 9 | 11 | 17 | 18 | 9 |
| **Hypothyroidism** | 45 | 31 | 35 | 67 | 54 |
| **Diabetes mellitus** | 16 | 10 | 13 | 12 | 27 |
| **Organomegaly** | 79 | 63 | 76 | 86 | 72 |
| **Splenomegaly ^a^** | 48 | 31 | 59 | 71 | 27 |
| **Hepatomegaly ^b^** | 58 | 23 | 45 | 47 | 27 |
| **Lymphadenopathy** | 27 | 42 | 35 | 75 | 63 |
| **Skin abnormalities** | 48 | 69 | 84 | 90 | 72 |
| **Hyperpigmentation** | 30 | 25 | 31 | 83 | 9 |
| **Glomeruloid angioma** | 21 | 23 | 68 | 35 | 27 |
| **Hypertrichosis** | 6 | 20 | 19 | 35 | 9 |
| **Nail changes** | 3 | 22 | NA | NA | NA |
| **Extravascular volume overload** | 58 | 70 | 81 | 88 | 72 |
| **Papilledema** | 12 | 30 | 24 | 64 | 27 |
| **Thrombocytosis** | 30 | 34 | 16 | 55 | 9 |

^a^ Defined as spleen length > 13cm.

^b^ Defined as a craniocaudal liver length (in the midclavicular line) greater than 16 cm and/or a transverse liver length (at the level of the upper poles of the kidneys) greater than 20 cm.
